# Enhancing Low-Light Images with Kolmogorov–Arnold Networks in Transformer Attention †

**DOI:** 10.3390/s25020327

**Published:** 2025-01-08

**Authors:** Alexandru Brateanu, Raul Balmez, Ciprian Orhei, Cosmin Ancuti, Codruta Ancuti

**Affiliations:** 1 Department of Computer Science, University of Machester, Manchester M13 9PL, UK; raul.balmez@student.manchester.ac.uk; 2 Faculty of Electronics, Telecommunications and Information Technologies, Polytechnic University Timisoara, 300223 Timisoara, Romania; ciprian.orhei@upt.ro

**Keywords:** image sensor restoration, low-light enhancement, Vision Transformer

## Abstract

Low-light image enhancement (LLIE) techniques improve the performance of image sensors by enhancing visibility and details in poorly lit environments and have significantly benefited from recent research into Transformer models. This work presents a novel Transformer attention mechanism inspired by the Kolmogorov–Arnold representation theorem, incorporating learnable non-linearity and multivariate function decomposition. This innovative mechanism is the foundation of KAN-T, our proposed Transformer network. By enhancing feature flexibility and enabling the model to capture broader contextual information, KAN-T achieves superior performance. Our comprehensive experiments, both quantitative and qualitative, demonstrate that the proposed method achieves state-of-the-art performance in low-light image enhancement, highlighting its effectiveness and wide-ranging applicability. The code will be released upon publication.

## 1. Introduction

Image sensors often face challenges in low-light conditions, such as noise and reduced contrast, which degrade image quality. Low-light image enhancement (LLIE) is a specialized area of computer vision that focuses on improving image clarity, contrast, and overall quality under such conditions, addressing issues caused by poor lighting and distortions. Such degradation not only results in subjectively unpleasant visual experiences but also impairs the performance of many CV systems. The goal of LLIE is to enhance visibility and contrast while simultaneously addressing the distortions inherent in dark environments.

Low-light conditions describe environments where illumination levels are insufficient for optimal visibility. However, defining precise theoretical thresholds to characterize low-light environments has proven challenging in practical applications. Consequently, there is no universally accepted standard for identifying or quantifying what constitutes low-light conditions [1].

Classical LLIE methods, such as gamma correction and histogram equalization, have limitations in effectively addressing low-light distortions. Traditional CV approaches attempt to improve upon these methods by considering illumination factors; however, they often introduce additional artifacts and imbalances in the restored images [1].

LLIE techniques have significantly progressed with Convolutional Neural Network (CNN) approaches, with solutions generally falling into two main categories. The first approach uses CNNs to directly map low-light images to their normal-light counterparts. While effective, this method often disregards human color perception and lacks theoretical interpretability. The second approach, inspired by Retinex theory [2], employs a more intricate multi-stage training pipeline. This method utilizes different CNNs for specific tasks, such as decomposing the color image, denoising the reflectance, and adjusting illumination. Although more consistent with theoretical models, this approach introduces considerable complexity and requires multiple training stages, posing significant challenges [3].

Although CNNs are highly effective at capturing local features, they often struggle with modeling long-range dependencies and adapting to broader contextual information. The self-attention mechanism introduced in the Transformer architecture [4] has partially addressed these challenges and has been adapted for image processing through Vision Transformers (ViTs) [4]. Recently, ViTs have been utilized in various LLIEs [5,6,7].

Kolmogorov–Arnold Networks (KANs) [8] are a recent advancement in machine learning, providing an innovative alternative to traditional Multi-Layer Perceptrons (MLPs). KANs leverage the decomposition of multivariate functions into univariate functions and linear combinations, enabling learnable activation functions on the edges (connections) between nodes. This design reduces computational complexity while enhancing performance, making KANs a promising solution for various deep learning (DL) applications.

While the specific advantages of KANs over MLPs are still being researched across different domains and scopes, their potential applicability to the LLIE domain is promising. LLIE inherently requires addressing both global context, such as overall brightness and contrast, and local context. Given KANs’ ability to approximate complex, non-linear functions and their expressive architecture, they align well with the intricate requirements of LLIE tasks. As highlighted in [9], KANs demonstrate enhanced capabilities for modeling complex transformations, both pixel-level precision and broader image-level characteristics.

In this work, we introduce a novel self-attention mechanism inspired by the Kolmogorov–Arnold representation theorem [10]. This theorem states that any multivariate continuous function can be expressed as a superposition of continuous functions and addition. In neural networks, KANs utilize this principle to break down multivariate functions into a series of univariate functions and linear combinations. Unlike traditional MLPs, which apply fixed activation functions to nodes and learnable weights to edges, KANs employ learnable activation functions on edges, offering greater flexibility. Drawing from these ideas and building upon our preliminary findings published in [11], our proposed attention mechanism adopts a similar approach to decompose multivariate functions into simpler, more manageable components.

**Figure 1:**
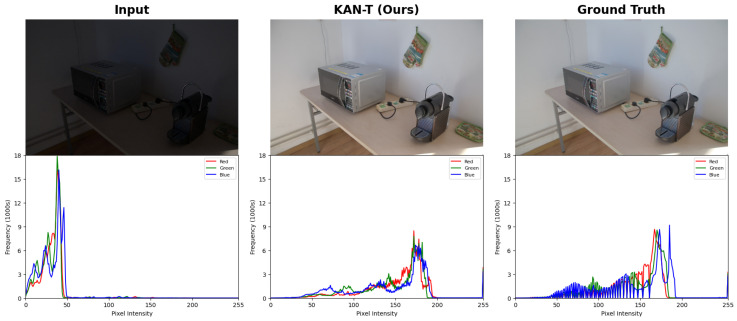
Visual presentation of our proposed method for LLIE.

Our primary contributions are outlined below:

- We develop a robust Transformer self-attention mechanism that significantly outperforms conventional channel-wise multi-headed self-attention methods. This advancement leads to improved model performance and more effective feature representation.
- We introduce a novel approach that integrates the principles of the Kolmogorov–Arnold representation theorem into attention mechanisms tailored for vision networks. This theoretical foundation enables more sophisticated and efficient feature map decomposition, enhancing the model’s capability to capture complex visual patterns.
- Our proposed method achieves superior results on various LLIE benchmarks. These advancements are validated through extensive quantitative and qualitative evaluations, demonstrating the effectiveness and reliability of our approach in enhancing images under challenging low-light conditions, as can be seen in Figure 1.

This paper is organized as follows: Section 1 introduces the research and highlights its significance. Section 2, titled “Related Work”, provides an overview of the LLIE domain. In Section 3, “Methods”, we describe the proposed model and its key components. Section 4 presents the results obtained on the LOL dataset [3]. Finally, this paper concludes with an ablation study in Section 5 and a discussion of future research directions in Section 6.

## 2. Related Work

Early LLIE methods focused on directly enhancing the contrast and brightness of images using techniques such as histogram equalization and gamma correction [1,12,13,14]. While these approaches are straightforward, they often fail to address the complexities introduced by low-light conditions, such as noise and artifacts.

Traditional CV methods based on Retinex theory [2,15,16,17] decompose an image into reflectance (color information) and illumination components. These methods improve image quality by adjusting illumination; however, they often neglect the noise and artifacts prevalent in low-light environments.

The advent of DL has transformed LLIE, with CNNs becoming central to image restoration tasks. LLNet [18] was the first deep-learning-based model for low-light enhancement, while EnGAN [19] introduced a single generator model to directly transform low-light images into normal-light versions. Wei et al. [3] proposed a CNN-based Retinex decomposition framework to enhance the illumination component and reconstruct a well-lit image. Despite their success, these approaches often involve complex, multi-stage training pipelines and are computationally intensive. Additionally, CNNs struggle to capture long-range dependencies, which limits their adaptability in diverse low-light scenarios.

Transformers, originally developed for machine translation [20], have recently gained prominence in image restoration tasks [21,22,23,24]. ViTs [4] excel at modeling long-range dependencies through self-attention mechanisms, surpassing CNNs in various image enhancement tasks. For LLIE, methods like UFormer [25] and Retinexformer [7] have emerged, leveraging Transformers for superior performance. UFormer adapts the U-Net framework [26] by replacing convolutions with Transformer blocks while preserving the hierarchical encoder–decoder structure with skip connections. Retinexformer employs illumination representations to model non-local interactions across regions with varying lighting conditions.

KANs [8] represent a recent innovation in machine learning, offering an alternative to traditional MLPs. By decomposing multivariate functions into univariate functions and linear combinations, KANs introduce learnable activation functions on the edges between nodes. This reduces computational requirements and enhances performance, making KANs a promising solution for various DL applications.

While classical and traditional LLIE methods laid the groundwork for enhancing low-light images, they often fall short in addressing the intricacies of real-world scenarios. DL approaches, especially those using CNNs, have achieved significant advancements but are limited by their inability to capture global dependencies. ViTs have emerged as a powerful alternative, offering robust solutions to LLIE challenges by leveraging self-attention mechanisms. Furthermore, the innovative design of KANs introduces a novel perspective for function decomposition, providing additional opportunities for improvement in LLIE methodologies.

## 3. Methods

### 3.1. Overall Framework

**Figure 2:**
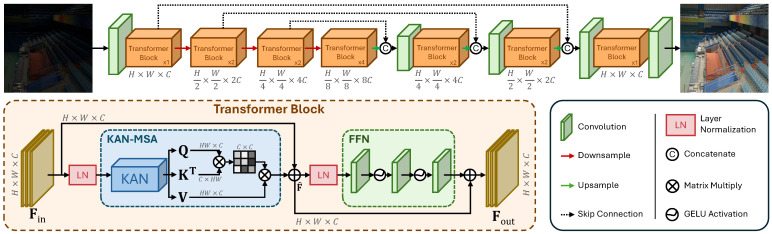
Overall framework of the proposed transformer. It presents a U-shaped arrangement with skip connections between same-level encoder–decoder blocks to help retain important dependencies.

Figure 2 illustrates the architecture of our proposed Transformer network, KAN-T, which employs a 3-level encoder-decoder structure. The input image is first passed through a conv1×1 layer that performs feature expansion, from H×W×3 to H×W×C. It is then processed by the encoder part of the network, which comprises Transformer blocks at different resolution levels, namely, H×W×C, $\frac{H}{2}\times \frac{W}{2}\times 2C$, and $\frac{H}{4}\times \frac{W}{4}\times 4C$. The encoder aims to transform the input image into an abstract internal representation that contains the key features in an image, which is then processed by the bottleneck section. The encoded feature map is then downsampled to $\frac{H}{8}\times \frac{W}{8}\times 8C$ and passed through the bottleneck of KAN-T, which utilizes four sequential Transformer blocks to enhance the internal feature representation. Then, the internal representation undergoes the decoding process, which consists of a suite of Transformer blocks at various levels, arranged symmetrically with respect to the encoder. The final H×W×C feature map undergoes a convolution to reduce the number of channels, producing the output image at H×W×3. KAN-T employs skip connections at corresponding encoder–decoder levels to help with detail preservation and feature enrichment.

### 3.2. Transformer Block

The Transformer block represents the main building block of KAN-T, and is used for its ability to perform advanced feature processing. As seen in Figure 2, the Transformer block consists of a Kolmogorov–Arnold Multi-headed Self-Attention (KAN-MSA) module, a Feed-Forward Network (FFN), and two Layer Normalization (LN) operations, while also employing residual connections between the two stages of self-attention and feature extraction. Given an input feature map $F_{\mathrm{in}}\in R^{H\times W\times C}$, we can formulate the first stage of the Transformer block processing as follows:

(1) $$\begin{array}{c} \hat{F}=\text{KAN-MSA}\left(\mathrm{LN}\left(F_{\mathrm{in}}\right)\right)+F_{\mathrm{in}},\;\hat{F}\in R^{H\times W\times C}, \end{array}$$

where $\hat{F}$ represents the layer-normalized, self-attended, and residually enhanced input feature map. Finally, the intermediate representation $\hat{F}$ undergoes the feature extraction stage of the Transformer block, where key features are enhanced, irrelevant features are discarded, and new features are discovered, and it can be expressed as

(2) $$\begin{array}{c} F_{\mathrm{out}}=\mathrm{FFN}\left(\mathrm{LN}\left(\hat{F}\right)\right)+\hat{F},\;F_{\mathrm{out}}\in R^{H\times W\times C}, \end{array}$$

where $F_{\mathrm{out}}$ is the output feature map resulting from the Transformer block processing.

### 3.3. Kolmogorov–Arnold Network Multi-Headed Self-Attention

The Multi-headed Self-Attention (MSA) module represents the most important component in Transformer architectures. It utilizes multiple attention “heads”, allowing the model to focus on different parts of the input simultaneously. Each head learns to capture distinct features or relationships in the data, enabling effective information representations from various subspaces. In encoder–decoder ViT architectures, it helps the model capture spatial hierarchies and long-range pixel dependencies, making it suitable in vision tasks where high resolution is key.

**Figure 3:**
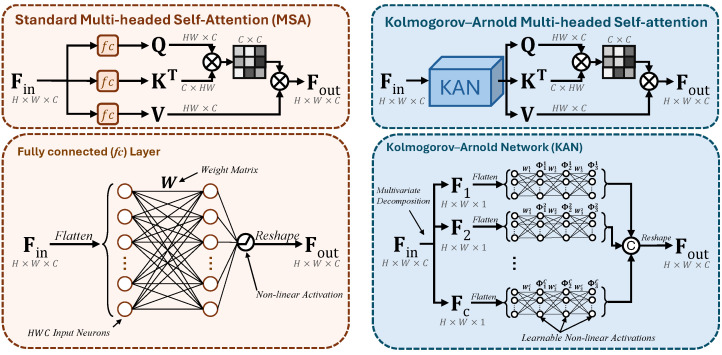
Overview of the standard MSA mechanism on the left, and our proposed KAN-based MSA on the right.

Given an input feature map $F_{\mathrm{in}}$, it first extracts the Query (Q), Key (K), and Value (V) components, then computes the attention map using Q and K, and finally applies this attention map to V, resulting in the self-attended version of $F_{\mathrm{in}}$. Standard MSAs utilize fully connected (*fc*) layers to obtain Q, K, and V. As seen in Figure 3, *fc* layers first flatten the input feature map and compute a weighted sum utilizing a weight matrix **W**, followed by a fixed non-linear activation and a reshaping operation. Given an input feature map $F_{\mathrm{in}}\in R^{H\times W\times C}$, this projection can be mathematically expressed as

(3) $$\begin{array}{c} Q=\sigma \left(F_{\mathrm{in}}W_{Q}+b_{Q}\right),\;K=\sigma \left(F_{\mathrm{in}}W_{K}+b_{K}\right),\;V=\sigma \left(F_{\mathrm{in}}W_{V}+b_{V}\right), \end{array}$$

where σ(·) is an activation function; $W_{Q}$, $W_{K}$, and $W_{V}$ are weight matrices; and $b_{Q}$, $b_{K}$, and $b_{V}$ are bias terms. While *fc* layers can model complex relationships by processing the entire multivariate input jointly, they may not efficiently capture univariate relationships within individual channels. Additionally, they can be computationally intensive due to the large number of parameters, especially for high-dimensional inputs.

To address these limitations, we introduce a KAN-based MSA mechanism inspired by the Kolmogorov–Arnold representation theorem [10], which states that any multivariate continuous function can be represented as a superposition of continuous univariate functions and addition. Our method also incorporates the aspect of learnable non-linearity, as seen in the original KAN formulation [8]. Given the input feature map $F_{\mathrm{in}}\in R^{H\times W\times C}$, we first formulate the multivariate decomposition by performing a channel-wise split as follows:

(4) $$F_{\mathrm{in}}=\left[F_{1},F_{2},\cdots ,F_{C}\right],\;F_{i}\in R^{H\times W\times 1},\;i=\left\{1,2,\cdots ,C\right\},$$

thereby reducing the problem of processing $F_{\mathrm{in}}$ at once—and therefore computing a multivariate function where each variable is a channel-wise component—to processing multiple single-channeled feature maps, enabling the model to capture more intricate and specific patterns in the data. Then, for each channel *i*, we process $F_{i}$ through a sequence of three fully connected layers with non-linear activations $\Phi_{j}^{i}$ as follows:

(5) $$\begin{array}{c} h_{1}^{i}=\Phi_{i}^{1}\left(W_{i}^{1}F_{i}+b_{i}^{1}\right),\;h_{i}^{2}=\Phi_{i}^{2}\left(W_{i}^{2}h_{i}^{1}+b_{i}^{2}\right),\;h_{i}^{3}=\Phi_{i}^{3}\left(W_{i}^{3}h_{i}^{2}+b_{i}^{3}\right),\;h_{i}^{3}\in R^{H\times W\times 3} \end{array}$$

By employing three sequential *fc*s, we allow the model to activate or deactivate certain neurons as they go through the $\Phi_{j}^{i}$ activations, ensuring learnable non-linearity. Finally, the results of the univariate processing are concatenated in a channel-wise manner to obtain $F_{\mathrm{out}}\in R^{H\times W\times 3C}$, which is then split three-way to obtain $Q,K,V\in R^{H\times W\times C}$. These are then reshaped to HW×C and used to produce the self-attended feature map $F_{\mathrm{out}}$ as

(6) $$F_{\mathrm{out}}=V\times \mathrm{softmax}\left(\frac{K^{T}\times Q}{\tau }\right),\;F_{\mathrm{out}}\in R^{HW\times C},$$

where τ is a learnable parameter used to balance attention scores, and $F_{\mathrm{out}}$ is later reshaped to H×W×C to preserve initial feature map dimensions.

### 3.4. Feed-Forward Network

The FFN is another key component of the Transformer block as it ensures in-depth feature extraction using the self-attended feature map. It follows a triple-convolution setup with Gaussian Error Linear Unit (GELU) [27] activations, denoted by ψ, and, given an input feature map $F_{\mathrm{in}}\in R^{H\times W\times C}$, is formulated as follows:

(7) $$F_{\mathrm{out}}=conv1\times 1\left(\psi conv3\times 3\left(\psi conv1\times 1\left(F_{\mathrm{in}}\right)\right)\right),\;F_{\mathrm{out}}\in R^{H\times W\times C},$$

where ψconv1×1 expands the feature map to H×W×4C to help with discovering new patterns, ψconv3×3 then performs high-resolution feature extraction by increasing the kernel size, and conv1×1 compresses the feature map back to original dimensions H×W×C.

### 3.5. Loss Function

To achieve precise reconstruction, we employ a composite loss function, denoted as L. Similar to other works like [28,29,30], our hybrid loss integrates multiple components to address various aspects of image quality, including pixel-level accuracy, structural integrity, and perceptual fidelity. The overall loss is formulated as

(8) $$L=L_{\mathrm{MAE}}+\alpha \cdot L_{\text{MS-SSIM}}+\beta \cdot L_{\mathrm{Perc}}$$

where α and β are hyperparameters that balance the contribution of each loss component.

Serving as the primary term in our loss function, the Mean Absolute Error (MAE) Loss $L_{\mathrm{MAE}}$ captures the average differences between the predicted image $\hat{I}$ and the ground truth image $I_{\mathrm{GT}}$. For a pixel at coordinates (x,y), this loss is defined as

(9) $$L_{\mathrm{MAE}}\left(x,y\right)=\frac{1}{N}\sum_{x,y}{∥\hat{I}\left(x,y\right)-I_{\mathrm{GT}}\left(x,y\right)∥}_{1}$$

The Multiscale Structural Similarity Index Measure Loss $L_{\text{MS-SSIM}}$ component evaluates the structural similarity [31] between the predicted and ground truth images across multiple scales. By assessing structural distortions, especially under challenging conditions like low-light scenarios, $L_{\text{MS-SSIM}}$ captures higher-level features that are crucial for maintaining the integrity of image structures. It is mathematically expressed as

(10) $$L_{\text{MS-SSIM}}=1-\prod_{m=1}^{M}{\left(\frac{2\eta_{m}\zeta_{m}+C_{1}}{\eta_{m}^{2}+\zeta_{m}^{2}+C_{1}}\right)}^{\delta_{m}}\times {\left(\frac{2\xi_{m}+C_{2}}{\kappa_{m}+\lambda_{m}+C_{2}}\right)}^{\epsilon_{m}}$$

Here, $C_{1}$ and $C_{2}$ are constants for luminance and contrast-structure stability, respectively, and *M* denotes the number of scales. For each scale *m*, $\eta_{m}$ and $\zeta_{m}$ represent the means of $\hat{I}$ and $I_{\mathrm{GT}}$, while $\kappa_{m}$ and $\lambda_{m}$ are their variances. The term $\xi_{m}$ stands for the covariance between $\hat{I}$ and $I_{\mathrm{GT}}$, and $\delta_{m}$ and $\epsilon_{m}$ are weights assigned to luminance and contrast-structure components.

Finally, the Perceptual Loss $L_{\mathrm{Perc}}$ [32] is used to incorporate feature-level supervision by leveraging a pre-trained VGG-19 network [33], denoted by Ψ. This loss measures the discrepancies between high-level feature representations of the predicted and ground truth images, facilitating the learning of meaningful internal representations, and is defined as

(11) $$L_{\mathrm{Perc}}\left(x,y\right)=\frac{1}{N}\sum_{x,y}{∥\Psi \left(\hat{I}\left(x,y\right)\right)-\Psi \left(I_{\mathrm{GT}}\left(x,y\right)\right)∥}_{1}$$

In this equation, Ψ denotes the VGG-19 feature extractor, and ${∥\cdot ∥}_{1}$ measures the absolute differences between the feature maps of the predicted and ground truth images.

By integrating these three loss components, our hybrid loss function effectively balances pixel-level accuracy, structural consistency, and perceptual quality, leading to enhanced performance in RGB image reconstruction tasks.

### 3.6. Implementation Details

We train our model on datasets such as LOL-v1 [3] and LOL-v2 [34], including both Real and Synthetic partitions, and evaluate it on the corresponding benchmarks.

Architecturally, our Transformer employs a framework with three encoder levels and three decoder levels, comprising [1,2,2] Transformer blocks at each level, respectively. The model uses [2,2,4] attention heads at the corresponding levels, and the FFN expansion rate η is set to 4. The attention dimensions are set as [24,48,96]. The loss function parameters are set as α=0.2 and γ=0.01.

Training is conducted on 256×256 patches with data augmentation techniques such as random cropping and random flipping, using a batch size of 2. We utilize the AdamW optimizer [35], with the parameters $\beta_{1}=0.9$ and $\beta_{2}=0.999$ and a weight decay of $1\times 10^{-4}$, over 150,000 iterations. The learning rate starts at $3\times 10^{-4}$ and is reduced to $1\times 10^{-6}$ via the cosine annealing schedule [36].

## 4. Results

In this section, we evaluate our proposed method against other state-of-the-art approaches. First, we visually assess the results obtained on the LOL dataset. Second, we measure the quantitative performance of various methods using the Peak Signal-to-Noise Ratio (PSNR) and the Structural Similarity Index Measure (SSIM) [31].

**Figure 4:**
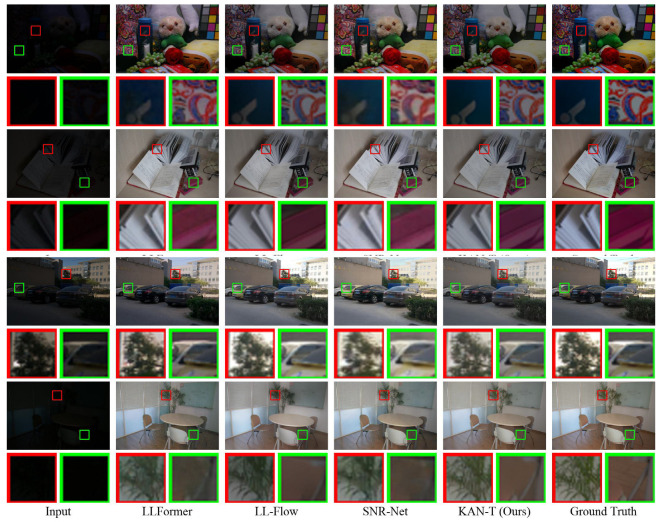
Comparison of qualitative results for different methods on LOL-v1 and LOL-v2-Real datasets. Red and green frames present zoomed-in regions for in-depth analysis.

**Qualitative Results.** Figure 4 presents qualitative comparisons of our model against several state-of-the-art methods. Approaches such as LLFormer [37] and LLFlow [38], while effective in improving brightness, often introduce unwanted lighting distortions, leading to overexposed or unevenly lit areas. Similarly, SNR-Aware [39] methods, although capable of denoising, frequently compromise the preservation of accurate color information, resulting in un-natural color reproduction. In contrast, our proposed KAN-T model demonstrates superior fidelity, closely matching the ground truth images. It achieves visually pleasing results that are both natural and richly detailed, preserving the subtle textures and true-to-life colors of the scene.

This balance is critical for real-world applications in domains such as surveillance, medical imaging, and autonomous driving, where the accurate reproduction of scene details under challenging lighting conditions is essential. KAN-T’s ability to maintain high visual fidelity, while effectively enhancing low-light images, underscores its superiority in tackling the complex challenges of LLIE. Furthermore, its robustness against unwanted distortions ensures reliability in scenarios where image quality directly impacts performance and decision making, further validating its applicability across diverse fields.

**Quantitative Results.** Table 1 presents the quantitative performance of various LLIE methods on the LOL-v1, LOL-v2-Real (LOLv2-R), and LOL-v2-Synthetic (LOL-v2-S) datasets. On LOLv2, KAN-T outperforms the previous state-of-the-art model, Retinexformer, by an average of 0.24 dB in PSNR while maintaining competitive SSIM values, showcasing the effectiveness of the proposed KAN-based attention mechanism. Notably, KAN-T secures top ranks across multiple metrics, demonstrating its robustness in handling diverse lighting conditions and complex scenarios in low-light image enhancement.

This performance is further complemented by KAN-T’s computational efficiency. While achieving superior results, it maintains a parameter count of only 2.80 M, significantly lower than competing methods such as LLFlow (37.68 M), LLFormer (24.55 M), and SNR-Aware (39.13 M). This reduced complexity makes KAN-T more suitable for real-time and resource-constrained applications without compromising quality. The model’s balanced approach to performance and efficiency highlights its potential for practical deployment in fields like surveillance, autonomous driving, and medical imaging, where both accuracy and speed are critical.

Overall, the results illustrate that KAN-T not only excels in quantitative metrics like PSNR and SSIM but also demonstrates scalability and adaptability to different datasets. Its superior performance on LOL-v2 and competitive results on LOL-v1 validate its robustness and make it a promising choice for advancing the state of the art in low-light image enhancement.

**Figure 5:**
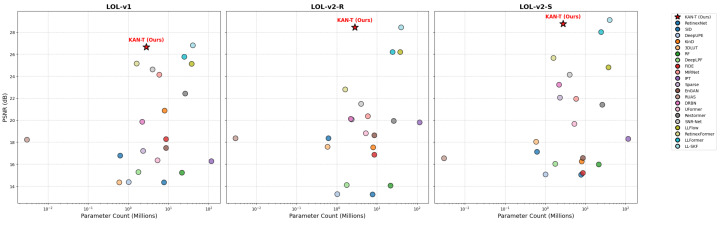
Parameter vs. PSNR performance across LOL-v1, LOL-v2-R, and LOL-v2-S datasets.

Figure 5 presents a comparative analysis of the PSNR performance of LLIE models presented in Table 1 on the LOL-v1, LOL-v2-R, and LOL-v2-S datasets against their respective parameter counts. This balance between high PSNR performance and efficient parameter usage demonstrates the strength and competitiveness of the proposed model.

## 5. Ablation Study

We conduct an ablation study on the LOL-v1 dataset to demonstrate the effectiveness of our proposed framework KAN-T, and utilize PSNR to measure performance, and number of parameters to evaluate complexity, where applicable.

We begin by evaluating the effectiveness of our proposed **QKV** extraction mechanism within the MSA framework, as detailed in Table 2a. The baseline model, which employs a standard MSA with fully connected (*fc*) layers, is the most lightweight, containing 1.29 million parameters. However, it yields a modest PSNR of 25.23 on the LOL-v1 validation set. Introducing our KAN-MSA with a depth of 1—where channel-wise processing is performed via a single *fc* layer in the KAN—results in a substantial improvement of 0.9 dB PSNR over the baseline. Further experimentation with increased depths of 3 and 5 demonstrates that a depth of 3 provides the highest PSNR gain of 1.43 dB, while a depth of 5 offers a smaller increase of 1.19 dB, indicating diminishing returns.

The effectiveness of our composite reconstruction loss L is substantiated through an ablation study presented in Table 2b. Starting with the foundational MAE loss ($L_{\mathrm{MAE}}$), which achieves a PSNR of 25.71, we observe that incorporating the Perceptual Loss ($L_{\mathrm{Perc}}$) alone enhances the PSNR to 26.21. This improvement highlights the significance of aligning high-level perceptual features in the reconstruction process. Similarly, integrating the MS-SSIM ($L_{\text{MS-SSIM}}$) with $L_{\mathrm{MAE}}$ results in a PSNR of 26.07, underscoring the role of structural consistency in achieving high-quality reconstructions. Most notably, the combination of all three loss components—$L_{\mathrm{MAE}}$, $L_{\mathrm{Perc}}$, and $L_{\text{MS-SSIM}}$—yields the highest PSNR of 26.66. This demonstrates the effect of integrating pixel-level accuracy, perceptual fidelity, and structural integrity, thereby validating the necessity of a hybrid loss function for optimal RGB image reconstruction performance.

## 6. Conclusions and Future Work

In this paper, we introduce an innovative attention mechanism based on Kolmogorov–Arnold Networks (KANs) and seamlessly integrate it into a Transformer architecture, which we designate as KAN-T, specifically designed for low-light image enhancement (LLIE). By leveraging the principles of the Kolmogorov–Arnold representation theorem within vision networks, we have developed a sophisticated feature map decomposition strategy. This approach incorporates learnable non-linearities through the application of multiple non-linear activation functions, enabling the model to capture and process complex visual patterns more effectively.

Leveraging KANs in LLIE tasks seems promising, especially if computational resources and training setups can accommodate their demands. However, future work could empirically validate the advantages of KAN architectures over MLPs in different domains.

Our comprehensive study demonstrates the successful adaptation of Kolmogorov–Arnold Networks to vision-based Transformer architectures, showcasing their superior performance compared with traditional fully connected (*fc*) layers. The KAN-T model achieves state-of-the-art results across various LLIE benchmarks, underscoring its efficacy in enhancing image quality under low-light conditions. Beyond addressing the inherent limitations of conventional Convolutional Neural Networks (CNNs) and standard Transformer models, our proposed framework establishes a new benchmark in the realm of low-light image enhancement. This advancement is validated through extensive quantitative metrics and qualitative assessments, highlighting the model’s ability to deliver exceptional performance and set a new standard in the field.

## Figures and Tables

**Table 1:** Results on LOL-v1 and LOL-v2 datasets and parameter counts of different models. **Red**, **blue**, and **green** metrics represent first, second, and third places, respectively.

Methods	LOL-v1		LOL-v2-R		LOL-v2-S		Param (M)
	PSNR	SSIM	PSNR	SSIM	PSNR	SSIM	
RetinexNet [3] BMVC ’18	16.77	0.462	18.37	0.723	17.13	0.798	0.62
SID [40] ICCV ’19	14.35	0.436	13.24	0.442	15.04	0.610	7.76
DeepUPE [41] CVPR ’19	14.38	0.446	13.27	0.452	15.08	0.623	1.02
KinD [42] MM ’19	20.87	0.799	17.54	0.669	16.26	0.591	8.03
3DLUT [43] TPAMI ’20	14.35	0.445	17.59	0.721	18.04	0.800	0.59
RF [44] AAAI ’20	15.23	0.452	14.05	0.458	15.97	0.632	21.54
DeepLPF [45] CVPR ’20	15.28	0.473	14.10	0.480	16.02	0.587	1.77
FIDE [46] CVPR ’20	18.27	0.665	16.85	0.678	15.20	0.612	8.62
MIRNet [47] ECCV ’20	24.14	0.842	20.36	0.782	21.94	0.846	5.90
IPT [22] CVPR ’21	16.27	0.504	19.80	0.813	18.30	0.811	115.31
Sparse [34] TIP ’21	17.20	0.640	20.06	0.816	22.05	0.905	2.33
EnGAN [19] TIP ’21	17.48	0.652	18.64	0.677	16.57	0.734	8.64
RUAS [48] CVPR ’21	18.23	0.720	18.37	0.723	16.55	0.652	0.003
DRBN [49] TIP ’21	19.86	0.834	20.13	0.830	23.22	0.927	2.21
UFormer [25] CVPR ’22	16.36	0.771	18.82	0.771	19.66	0.871	5.29
Restormer [5] CVPR ’22	22.43	0.823	19.94	0.827	21.41	0.830	26.13
SNR-Net [39] CVPR ’22	24.61	0.842	21.48	0.849	24.14	0.928	4.01
LLFlow [38] AAAI ’22	25.13	**0.872**	26.20	**0.888**	24.81	0.919	37.68
Retinexformer [7] ICCV ’23	25.16	0.845	22.80	0.840	25.67	**0.930**	1.61
LLFormer [37] AAAI ’23	**25.76**	0.823	**26.20**	0.819	**28.01**	0.927	24.55
LL-SKF [50] CVPR ’23	**26.80**	**0.879**	**28.45**	**0.905**	**29.11**	**0.953**	39.91
KAN-T (Ours)	**26.66**	**0.854**	**28.45**	**0.884**	**28.77**	**0.939**	2.80

**Table 2:** Ablation studies on QKV extraction methods and loss functions. **Red** and **blue** metrics represent best and second-best results, respectively.

(a) Comparison of QKV Extraction Methods in MSA			(b) Impact of Different Loss Functions on Performance			
QKV	Param. (M)	PSNR	$L_{\mathrm{NAE}}$	$L_{\mathrm{Perc}}$	$L_{\mathrm{MS-SSIM}}$	PSNR
*fc*	1.29	25.23	✓			25.71
${\mathrm{KAN}}_{d=1}$	1.79	26.13	✓	✓		**26.21**
${\mathrm{KAN}}_{d=3}$	2.80	**26.66**	✓		✓	26.07
${\mathrm{KAN}}_{d=5}$	3.81	**26.42**	✓	✓	✓	**26.66**

## Data Availability

The original contributions presented in this study are included in the article. Further inquiries can be directed to the corresponding authors.

## Abbreviations

The following abbreviations are used in this manuscript:

CV	computer vision
CNN	Convolutional Neural Network
DL	deep learning
FFN	Feed-Forward Network
GELU	Gaussian Error Linear Unit
KAN	Kolmogorov–Arnold Network
KAN-MSA	Kolmogorov–Arnold Multi-headed Self-Attention
LLIE	low-light image enhancement
LN	Layer Normalization
MAE	Mean Absolute Error
MLPs	Multi-Layer Perceptrons
MSA	Multi-headed Self-Attention
PSNR	Peak Signal-to-Noise Ratio
SSIM	Structural Similarity Index Measure
ViTs	Vision Transformers
